# Coating bacteria for anti-tumor therapy

**DOI:** 10.3389/fbioe.2022.1020020

**Published:** 2022-09-15

**Authors:** Jiahui Wang, Ning Guo, Weiliang Hou, Huanlong Qin

**Affiliations:** ^1^ Department of Gastrointestinal Surgery, Shanghai Tenth People’s Hospital, Tongji University, Shanghai, China; ^2^ Shanghai Key Laboratory for Nucleic Acid Chemistry and Nanomedicine, Institute of Molecular Medicine, Renji Hospital School of Medicine, Shanghai Jiao Tong University, Shanghai, China

**Keywords:** tumor therapy, tumor targeting, coating, bacteria, materials

## Abstract

Therapeutic bacteria have shown great potential on anti-tumor therapy. Compared with traditional therapeutic strategy, living bacteria present unique advantages. Bacteria show high targeting and great colonization ability in tumor microenvironment with hypoxic and nutritious conditions. Bacterial-medicated antitumor therapy has been successfully applied on mouse models, but the low therapeutic effect and biosafe limit its application on clinical treatment. With the development of material science, coating living bacteria with suitable materials has received widespread attention to achieve synergetic therapy on tumor. In this review, we summarize various materials for coating living bacteria in cancer therapy and envision the opportunities and challenges of bacteria-medicated antitumor therapy.

## Introduction

With the rapid development of modern medical technology and industry, the average human lifetime is gradually extended (Vaupel et al., 2021). However, the treatment of tumor still is a global problem, despite that some mild tumor could be surgically excised. The improvement of symptoms, survival quality and period are currently the main purpose of tumor therapy (Wyld et al., 2015). Radiotherapy is the common therapy strategy for antitumor, while the insufficient tissue penetration and non-targeting limit its widespread application (Jain, 1998; Minchinton and Tannock, 2006; St-Jean et al., 2008; Grierson et al., 2017). Chemotherapy is an effective way to suppress the growth and spread of tumor with chemical drugs throughout the whole body, but its non-specificity on tumor cells could cause damage to normal tissues (Pérez-Herrero and Fernández-Medarde, 2015). Surgery could not completely clear metastatic cancer cells with the recurrent risk (Wyld et al., 2015). Chimeric antigen receptor T (CAR-T) cell therapy is regarded as an effective solution for relapsed or refractory tumors, due to high tumor targeting (Curran et al., 2012; Sadelain et al., 2013; Mirzaei et al., 2016). Potential side effects restrict the clinical application of CAR-T cell therapy, such as B cell abnormalities (Marofi et al., 2021). New therapy strategy with high tumor targeting, low side effect and good effect is needed for antitumor treatment.

Bacteria therapy could be a promising strategy on tumor treatment (Dang et al., 2001; Silva-Valenzuela et al., 2016). The hypoxic and nutrient-rich tumor microenvironment is uniquely attractive to bacteria (Nguyen and Min, 2017). In the early 19th centuries, Dr. Busch firstly noticed that patients with malignant tumors improved after being infected with *Streptococcus pyogenes* (*S. pyogenes*). In the mid-19th century, Coley (1910) found that people with neck cancer recovered from infection of erysipelas. Then Coley tried to treat tumors with inactivated bacteria, such as *S. pyogenes* and *Serrati amarcescens* and established the foundation of bacterial therapies on cancer (Richardson et al., 1999). In recent years, people find various bacteria with good tumor-targeting property, including *Salmonella* (Pawelek et al., 1997), *Escherichia*, *Clostridium* (Malmgren and Flanigan, 1955; Minton, 2003), *Bifidobacterium* (Kohwi et al., 1978), *Caulobacter*, *Listeria* (Pan et al., 1999; Kim et al., 2009), Proteus (Arakawa et al., 1968), and *Streptococcus* (Maletzki et al., 2008). *Caulobacter crescentus* (*C. crescentus*) as a Gram-negative non-pathogenic bacterium presented tumor suppressive effects in unmodified form (Bhatnagar et al., 2006). After non-tumorigenic activity in mouse models of transplantable tumors, the prolonged survival and reduced tumor mass of *C. crescentus* group presented better antitumorigenic activity in mouse models of lung tumor, breast tumor and leukemia tumors than saline controls. These results suggest that *C. crescentus* may be a safe bacterial immunomodulator for tumor treatment (Bhatnagar et al., 2006).

The ability to induce and amplify antigen-specific immune responses has been considered a potentially valuable tool on the treatment of cancer. Most cancer vaccines induce cytotoxic T lymphocyte (CTL) responses to tumor-associated antigens (TAA). An attenuated vaccine against *Listeria monocytogenes* (*L. monocytogenes*) eradicated metastases and the entire primary tumor of breast cancer in mice by TAA-specific CTL-mediated cytolysis to kill tumor cells (Kim et al., 2009). The vaccine mode of action of Listeria provides a new direction in bacterial research in targeting metastatic breast cancer. Further study found that *L. monocytogenes* could serve as an effective vehicle for tumor-specific antigen targeting (Kim et al., 2009). The engineered *L. monocytogenes* expressing tumor-specific antigen induced primary tumor regression and identified pulmonary metastases by parenteral immunization in murine model of melanoma B16F10 (Pan et al., 1999). The non-pathogenic parthenogenic anaerobic bacterium *Salmonella* can specifically target tumor sites to regulate immune response (Pawelek et al., 1997; Lee et al., 2005; Zhao et al., 2005; Forbes, 2010; Chang and Lee, 2014). The modulation of the antitumor effect of *Salmonella* encapsulated with polyallylamine hydrochloride not only greatly enhanced antitumor activity but also maintained tumor targeting (Lee et al., 2017).

In recent years, biomaterials have been used to decorate bacteria for achieving gastrointestinal protection and synergetic treatment on tumor based on its biodegradability, biocompatibility and immunomodulatory activity (Lee et al., 2013; Zhu et al., 2013). Feng et al. (2020) found that bacteria can be temporarily inactivated by confining them individually in an intact polymer coating. Bacteria are intelligently restored to vital activity after shedding, thus achieving targeted release of bacterial drugs. This approach greatly improves the bioavailability as well as the effectiveness and stability of bacteria during *in vivo* delivery, providing an important means to prepare bacterial-mediated smart biologics for tumor therapy. This paper reviews biomaterial styles and synergetic strategy for coating bacteria on tumor therapy in recent years. We hope this review could enlighten researcher on bacteria-mediated tumor therapy and guide more biomaterials used on bacterial coating.

## Materials for coating bacteria

Bacteria therapy offers a new perspective on anti-tumor treatment, but the instability and biosafety of living bacteria limit its clinical application (Sarotra and Medhi, 2016). Enteric bacteria can be directly transported to the host *via* intravenous injection, oral administration, or anal perfusion (Wu et al., 2013; West et al., 2020). Gavage and oral administration are considered as the most convenient and widely applicated method for bacteria delivery compared with anal perfusion considering the low patient compliance and local delivery (Zaloga, 2006).

The low pH environment, proteolytic enzyme, and high concentration of bile salts in gastrointestinal tract (GI tract) could significantly reduce the activity and therapeutic effects of oral bacteria (Evans et al., 1988; Cook et al., 2012; Sohail et al., 2018). The limitation restricts the use of oral bacteria. An effective approach of bacteria therapy is coating bacteria with suitable materials, hence achieving protection and controlled release of living bacteria (Prakash and Jones, 2005). Obviously, these materials should be acid-proof, safe, mild, and automatically degraded under certain conditions. The combination of biomaterials and encapsulation technologies could raise the efficiency of oral delivery and decrease the side effects of bacteria.

**FIGURE 1:**
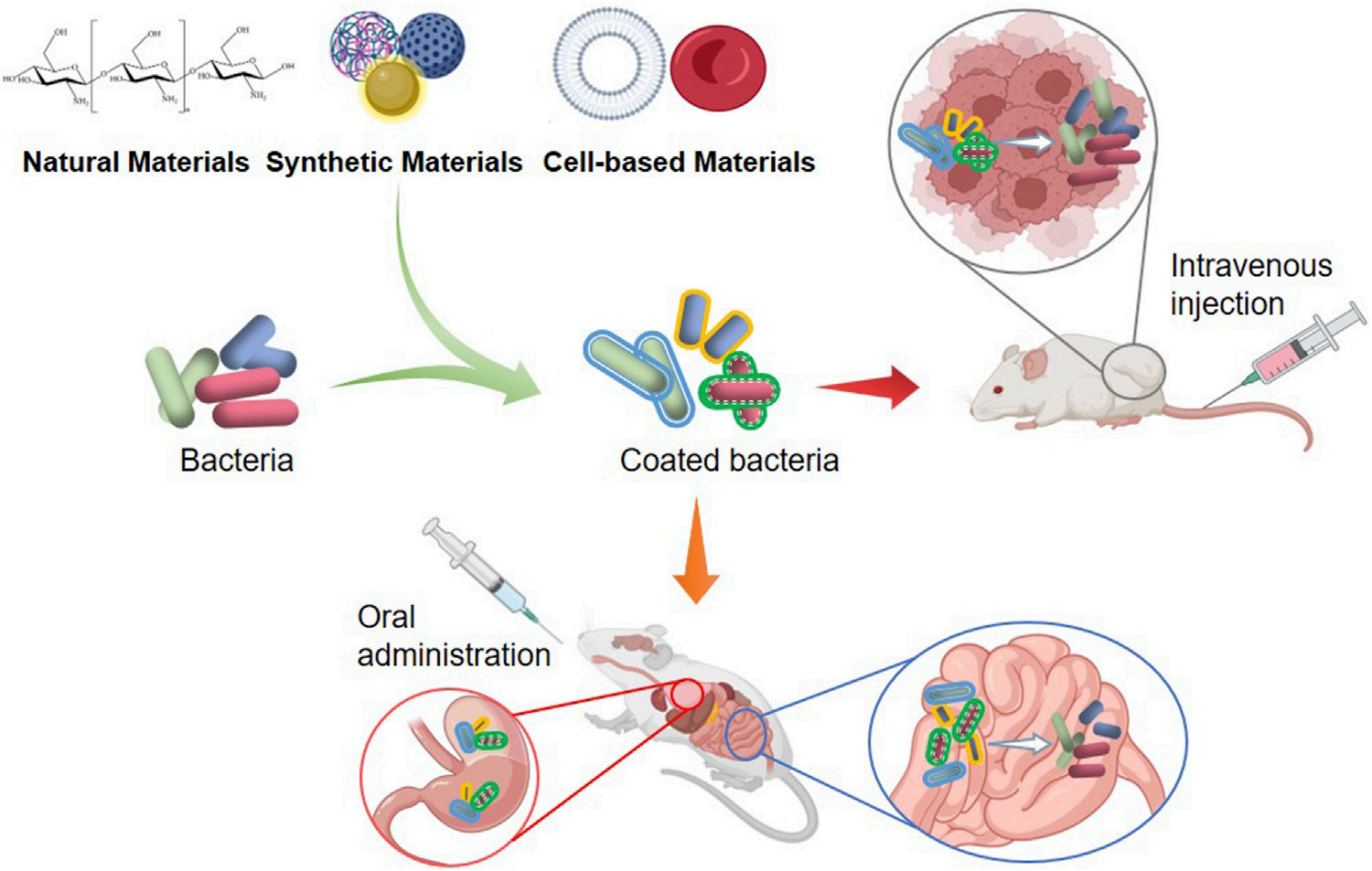
Typical materials for the delivery of bacteria.

Bacteria-medicated cancer therapy has made remarkable progress in recent years. The combination of bacteria with suitable materials could increase bacterial tumor-colonization and offset the shortage of drug supply into intra-tumoral regions, hence reducing side effects and improving antitumor efficacy (Felfoul et al., 2016; Lou et al., 2021). Drug-loaded bacteria could preferentially translocate to tumor stroma after intravenous administration and selectively release the drugs in response to the tumor microenvironment (Li et al., 2021). At present, a variety of biological materials have been used to encapsulate bacteria for tumor treatment, including natural materials, synthetic materials, and cell-based materials (Figure 1).

### Natural materials

Up to now, plenty of studies have demonstrated that a variety of natural polymers are suitable for coating bacteria, such as polysaccharides, Eudragit, proteins, poly (amino acids), and lipids (Wee and Gombotz, 1998; Li et al., 2016; Yeung et al., 2016; Dafe et al., 2017; Li et al., 2020).

Alginate comprised with a linear polysaccharide consisting of 1,4’- linked β-D-mannuronic acid and α-L-gluconic acid residues originating from microbes (e.g., *Pseudomonas*) or brown algae, is particularly suitable to encapsulate bacteria due to its nontoxicity and mild gelling conditions (Zhou et al., 1998; Rinaudo, 2008). Alginate can form an “egg box structure” between four G resides by contacting with divalent metal ions (e.g., Ca^2+^, Cd^2+^, Zn^2+^) (Stokke et al., 1997; Wahab et al., 1997; Cook et al., 2012), because of the carboxylic acid groups on both monomer molecules (Draget et al., 1994). The property of alginate has been exploited to produce microcapsules by an extrusion process. The alginate solution is dropped into cationic propyl gallate (commonly calcium chloride) gelling into a microcapsule. The size of the microcapsules formed by the external gel depends on the droplet size formed in an extrusion process, which is typically between tens of microns and millimetre size (Sun and Griffiths, 2000; Chandramouli et al., 2004; Ding and Shah, 2009).

Therefore, alginate is also a common tool of intestinal delivery vehicles. Numerous studies have shown that coating *lactobacillus* with alginate gel can help them resist gastric acid and ensure that enough living bacteria into the small intestine (Adhikari et al., 2000; Sultana et al., 2000; Pan et al., 2013). Interestingly, Lee and Heo, 2000) found that the survival rate of living bacteria elevated with increasing the alginate concentration. In addition, alginate could enhance bacterial resistance to antibiotics by fabricating biomimetic biofilm to entrap probiotics crossing-link with calcium ions by electrospray (Li et al., 2018). The application of alginate is restricted due to its instability, uncontrollable swelling, and fragility (Kim et al., 2014). Diffusion-gelled alginate degrades with exposure to biological buffers for a long time both *in vivo* and *in vitro* (Peirone et al., 1998; De Vos et al., 1999). The main reason could be the gel dissolution caused by the exchange of calcium and monovalent sodium ions (LeRoux et al., 1999; Van Raamsdonk and Chang, 2001). Therefore, many new strategies are attempted to strengthen the stability of alginate. For example, the co-coating of alginate and chitosan on the surface of bacteria remarkably elevated the stability and survival rate of bacteria (Wee and Gombotz, 1998; Călinoiu et al., 2019). Lin et al. (2008) have found that alginate-chitosan-alginate (ACA) microcapsules could entrap living bacteria to achieve ascendant chemical stability of bacteria in stimulated-gastric fluid *in vitro*. *In-vivo* studies proved that ACA microcapsules were more resistant to GI enzyme degradation than alginate-poly-lysine-alginate microcapsules.

Chitosan as a natural cationic polysaccharide is also considered as a coating material for bacteria due to its biodegradability, low toxicity, and biocompatibility (Lai and Lin, 2009; Netsomboon and Bernkop-Schnurch, 2016). Cook et al. (2011) found that *Bifidobacterium breve* coated with chitosan and alginate showed more tolerant to the GI tract than that by single alginate coating, because that chitosan could stable alginate microcapsules and maintain the stability of probiotics in the stomach. Cationic chitosan and anionic alginate could repeatedly encapsulate bacteria through electrostatic interaction (Anselmo et al., 2016). The layer-by-layer coating technique greatly improved the viability and stability of oral bacteria in the GI tract (Lin et al., 2008; Anselmo et al., 2016). In addition to chitosan and alginate, other materials also are used to encapsulate living bacteria applicated in the biomedical field, such as poly-L-lysine (Krasaekoopt et al., 2004), protamine (Mei et al., 2014), starch (Saberi-Rise and Moradi-Pour, 2020) or gelatin (Tu et al., 2015; Pour et al., 2019).

Poly-L-lysine (PLL), natural cationic polymers, could complex with alginate to form microcapsules *via* electrostatic attraction. Chen et al. (2005) designed alginate-poly-L-lysine-alginate (APA) microcapsules for oral administration of *Lactobacillus plantarum*. The APA microcapsules could maintain morphological stability under a simulated stomach condition, but failed to retain structural integrity after long-term exposure in a simulated gastro-intestinal medium. To enhance the stability of coating bacteria in the GI tract, Ouyang et al. (2004) prepared a novel multilayer APPPA (alginate-PLL-pectin-PLL-alginate) system to coat intestinal bacteria and showed better stability than APA microcapsules in simulated gastrointestinal fluid.

**FIGURE 2:**
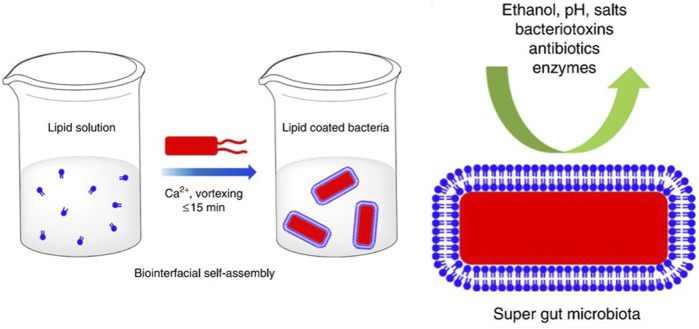
Schematic diagram of lipid membrane-coated bacteria *via* bio-interfacial self-assembly.

Phospholipids, the main components of cell membranes, are often used as delivery carriers for drugs or small molecules because of their biocompatibility, easy modification, low immune response and biodegradability (Matthay et al., 1984). Chowdhuri et al. (2016) used liposomes encapsulating *Escherichia coli* (*E. coli*) by the inverse-emulsion way and assessed the effect of liposomes on bacteria activity and viability. The inverse-emulsion method has been reported to coat efficiently biological macromolecules, such as proteins and living cells (Zhu H. et al., 2018). Cao et al. (2019b) utilized lipids to generate a myriad of super gut microbes by bio-interfacial supramolecular self-assembly, which not only improved the bioavailability of oral bacteria but also maintained the bioactivity (Figure 2).

**FIGURE 3:**
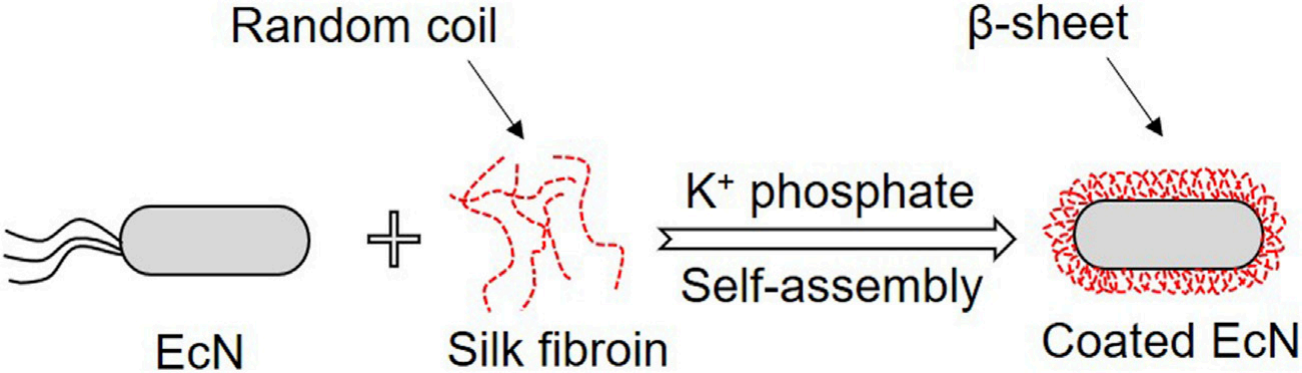
Schematic diagram of decorating bacteria with medicative silk fibroin by self-assembly.

Proteins are another important group of polymers for the encapsulation of bacteria due to their amphiphilic nature (Kim et al., 2016). The most common proteins used for encapsulation of probiotics include gelatin (Paula et al., 2019), whey protein (Yoda et al., 2015), and so on. Silk fibroin from a natural protein of silk-worm cocoon has great biocompatibility, biodegradability, non-immunogenicity, and mechanical robustness (Keten et al., 2010; Rockwood et al., 2011; Zhu Y. et al., 2018; Shi et al., 2019). Silk fibroin nanoparticles could specifically target inflammation sites and damaged intestinal tract, hence assisting with delivering drugs to inflamed tissues (Lamprecht et al., 2001; Gobin et al., 2006; Fathi et al., 2019). Silk fibroin could self-assemble on the surface of bacteria by transforming beta-sheet conformation from a random coil to form the core-shell structure for bacteria delivery (Figure 3) (Hou et al., 2021).

### Synthetic materials

**FIGURE 4:**
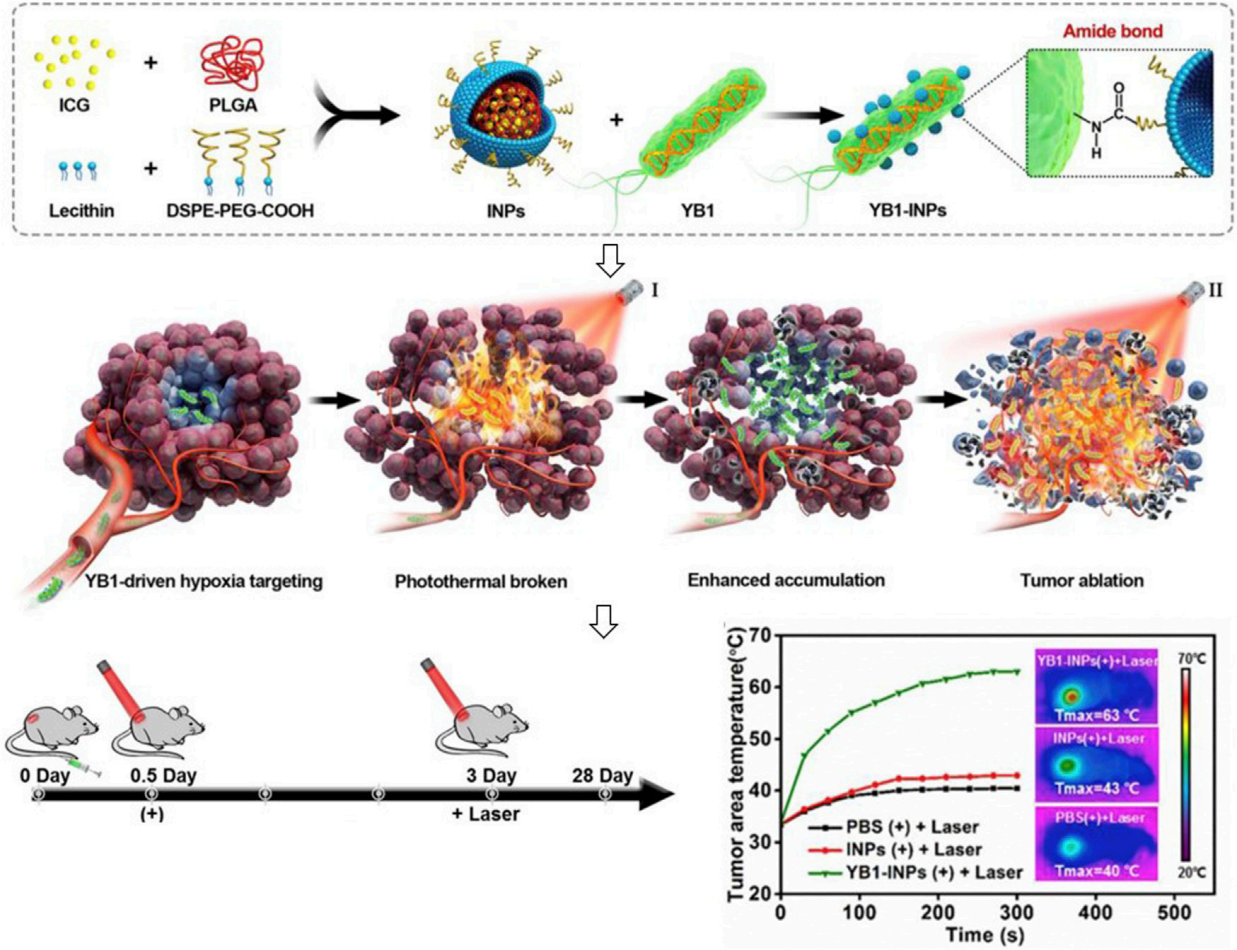
Encapsulation of indocyanine green-loaded nanoparticles on the surface of bacteria for tumor therapy.

With the development of encapsulation technology and materials science, synthetic materials also are used for coating living bacteria based on their tumor targeting, tumor tissue penetration, and anti-tumor effects. Chen et al. (2019) prepared indocyanine green-loaded nanoparticles and attached them to the surface of a genetically modified *Salmonella Typhimurium* YB1 through amide bonds to create a biotic/abiotic cross-linked system for large solid tumor precision therapy (Figure 4). This system showed stable and efficient photothermal killing ability after intravenous injection and completely eliminated large solid tumors. Taherkhani et al. (2014) proposed that combining carboxyl-modified drug-carrying nanoliposomes with amino groups on the surface of magnetotactic bacteria MC-1 could deeply penetrate hypoxic tumor sites by the external magnetic field. Doxorubicin (DOX) conjugated to *E. coli* Nissle 1917 (EcN) by acid-labile linkers of cis-Aconitum anhydride can release under an acidic environment and achieve directly anticancer drug accumulation (Xie et al., 2017).

Zhang et al. (2018) reported a heat-sensitive drug oral-delivery system in which thermally-sensitive programmable bacteria expressing therapeutic protein TNF-α were decorated with bio-mineralized gold nanoparticles. The engineered bacteria could reach the tumor regions through the GI tract after oral administration. Irradiating tumor sites with near-infrared light, gold nanoparticles could induce the expression of TNF-α from the engineered bacteria, hence inhibiting the growth of tumor cells.

Spore, the dormant life forms of bacteria, could enclose drugs and improve the bioavailability due to the resistance to the acidic enzyme-rich digestive tract. Spore loading with a chemotherapy drug DOX was modified with deoxycholic acid to create anti-tumor nanoparticles. The nanoparticles could effectually protect DOX in the GI tract and enhance the accumulation of DOX in tumor regions (Song et al., 2019).

### Cell-based materials

**FIGURE 5:**
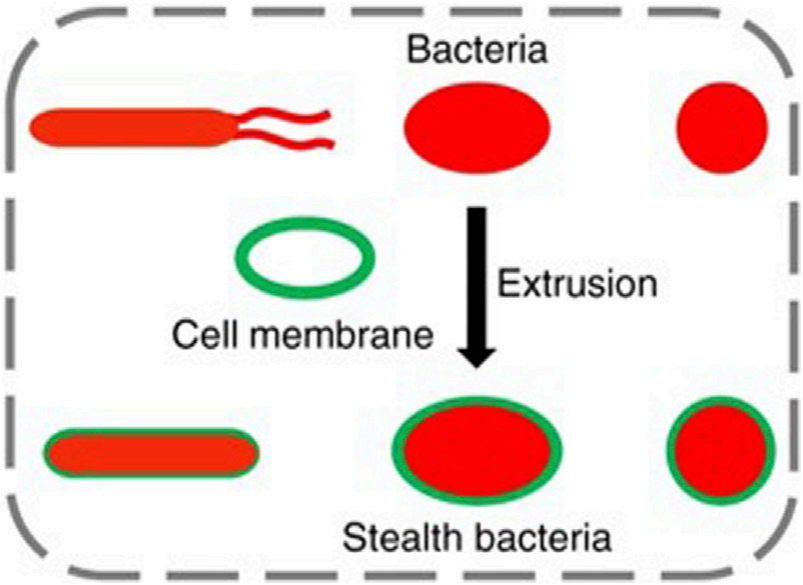
Schematic illustration of extruding bacteria with cell membranes.

Except for natural materials and synthetic materials, cell-based materials have been applied in the field of nano and micro motors considering their attractive properties such as biocompatibility and low immunogenicity. Red cell membrane-coated nanoparticles could prolong the circulation time of particles *in vivo* (Hu et al., 2011), and (Kulkarni et al., 2011) platelet membrane-coated nanoparticles could target specific tumor tissues and enhance the ability of injured sites colonization (Wang et al., 2019). The cell membrane is usually stripped from cells as the materials to decorate bacteria. For red blood cells, cells were first separated from the whole blood and the intracellular components were removed by hypotonic treatment. The hollowed-out red blood cells are then washed and extruded from the porous membrane to form small vesicles derived from the erythrocyte membrane (Luk and Zhang, 2015). Cao et al. (2019a) extruded erythrocyte membrane with EcN to obtain cell membrane-coated bacteria (CMCB). The CMCB achieved lower immunogenicity, inherent bioactivities and blood reservation up to 48 h after injection (Figure 5). Alapan et al. (2018) combined *E. coli* MG1655 with red blood cells to wrap doxorubicin and superparamagnetic iron oxide nanoparticles *via* biotin-avidin affinity, hence facilitating the delivery of drugs.

Except for drug delivery, cell membrane coating could also be applied to detoxification of pathogenic virulence factors (Hu et al., 2013a) and anti-virulence vaccination (Hu et al., 2013b) to treat bacterial infections led by *Staphylococcus aureus*, *Enterobacteriaceae*, and others. Cell membrane coating shows great potential for targeted delivery of therapeutic agents and bacteria to reduce undesirable off-target effects, since that a variety of targeting ligands and cell membranes can achieve cell-specific binding and uptake. The encapsulation of living bacteria by materials maximizes the survival rate and bioavailability of oral administration in the GI tract, and provides low immunogenicity of bacteria in the blood. However, bacteria-mediated bio-therapy is mainly based on animal models and the potential challenges should be solved before clinical application.

In brief, it is difficult for natural materials to be modified simultaneously while maintaining great bioavailability. Reversely, synthetic materials are very versatile. Synthetic materials can be designed with different functions according to the requirement of drug administration. However, most synthetic materials could be low bioavailability to the delivering bacteria or the delivered animals, which limits their application in clinic (Pan et al., 2021). Cell-based materials present high safety and long systemic circulation *in vivo*, while the specifical source of each cell membrane could impact on the commonality of cell-based materials. Therefore, natural materials or cell-based materials modified with synthetic materials could endow synergetic therapy with high bioavailability and low immunogenicity, which will accelerate the application of bacteria-mediated therapy in clinic.

## Bacteria-mediated antitumor therapy

**FIGURE 6:**
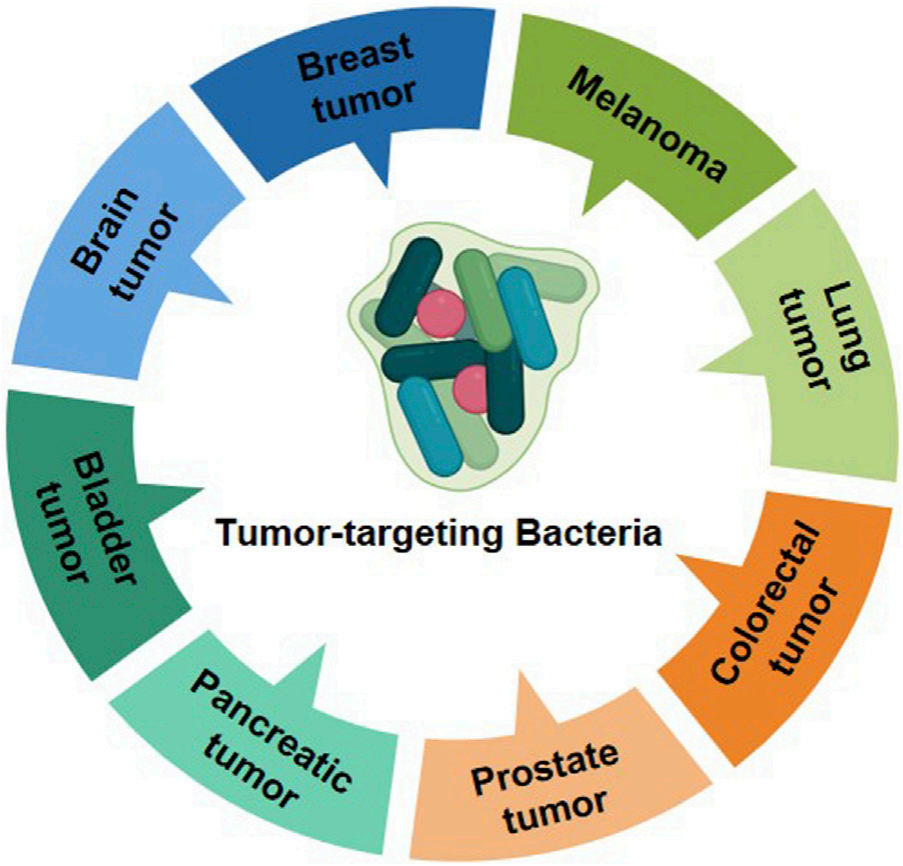
Bacteria mediate antitumor therapy.

Bacterial therapy in oncology could date back at least 150 years, because of the unique ability and easily manipulated genes of bacteria (Kulp and Kuehn, 2010). Some bacteria have well tumor colonization as facultative or strict anaerobes due to the hypoxic microenvironment on tumor, such as *E. coli* and *Salmonella typhimurium*. Tumor targeting bacteria have been applied on various tumors for diagnosis, imaging and treatment with or without functional decoration (Figure 6).

### Bacteria therapy on breast cancer

Breast cancer is the most common cancer among women worldwide with low survival rate (Alberg and Singh, 2001; Althuis et al., 2005). Current treatment options for metastatic cancer include surgery followed by chemotherapy or radiation therapy and/or adjuvant therapy (Scart et al., 2002). Tumor targeting bacteria could assist in the delivery of chemotherapy or radiation drugs to improve antitumor therapy. Zhang et al. (2018) constructed nanoscale microcells by genetically engineered ECN as a carrier for the targeted delivery of chemotherapeutic drugs to tumor hypoxic zone. The drug-carrying microcells showed significant inhibition on the growth of breast cancer without any significant toxicity. Raman et al. (2021) developed a *Salmonella* vector with controlled drug synthesis and cellular invasion and achieved effectively reduced tumor growth and metastasis. *Salmonella*-based protein delivery shows a safe and effective treatment for tumors to provide new therapy for untreatable cancers.

### Bacteria therapy on melanoma

**FIGURE 7:**
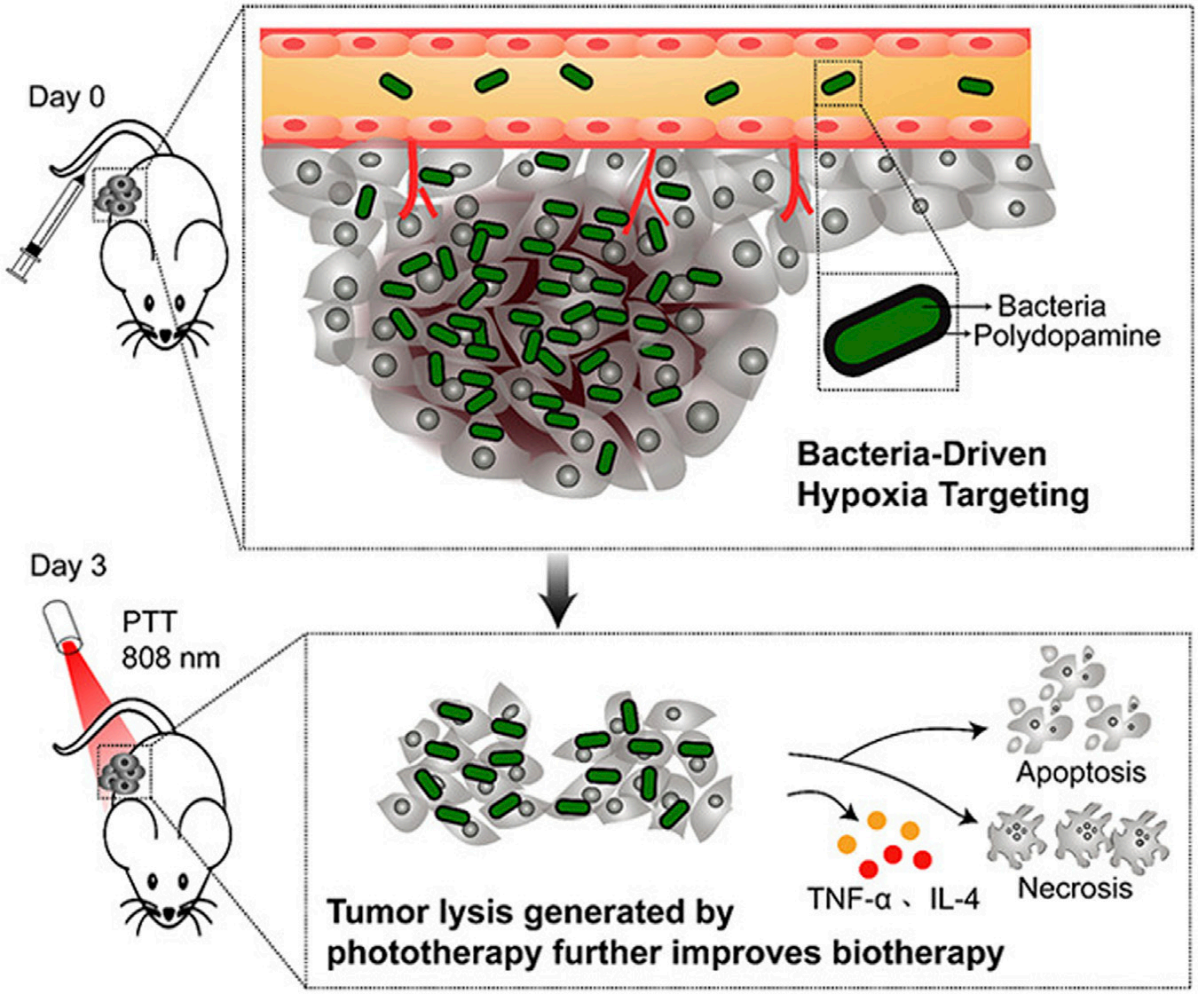
Bacteria combined with polydopamine-mediated photothermal therapy on melanoma.

The facultative anaerobe *Salmonella* strain VNP20009 prefers to colonize hypoxic areas of tumor core (Chen et al., 2017) and necrotic tumor tissue (Chang and Lee, 2014). The attenuated strain VNP20009 can carry exogenous gene expression plasmids to target tumors and express exogenous proteins specifically in tumor tissues. Study showed that VNP20009 combined with photothermal therapy can achieve higher specificity and anti-tumor effects. The photothermal agent converts the incident light into heat in the irradiated target tissue to kill the surrounding tumor cells (Liu et al., 2013; Fan et al., 2018; Jung et al., 2018; Vankayala and Hwang, 2018). Chen et al. (2018) encapsulated polydopamine on the outer layer of VNP20009 to induce apoptosis and necrosis of melanoma cells in mouse model, thereby inhibiting tumor growth (Figure 7). The antitumor of different Salmonella strains was investigated (Avogadri et al., 2008; Crull et al., 2011; Kocijancic et al., 2017). A nutrient-deficient *Salmonella enterica* with aroA mutant was identified to possess immunostimulatory potential and hence achieved the inhibition of melanoma cells growth and the improved survival rate of mice (Johnson et al., 2021).

Based on the metabolic characteristics of bacteria, Shi et al. (2022) showed that the metabolism of oncolytic bacteria with synthetic photosensitizer-label could specifically clear anaerobic regions of tumors in concert with photodynamic therapy. The new functionalized lysing bacterium combined with the bio-photodynamic-immunotherapy promised minimally invasive removal of malignant melanoma, hence providing a new tool for post-operative recurrence prevention. These studies have shown great potential of bacterial therapy on the treatment of melanoma to provide a more effective and safe treatment strategy.

### Bacteria therapy on colorectal tumor

Colorectal cancer (CRC) is the third most common cancer and the second leading cause of cancer deaths in the United States (Goldberg et al., 2007; Jemal et al., 2008). Except for traditional radiotherapy and chemotherapy, bacterial therapy was used on the CRC treatment long ago and is booming with the development of biomedical technology and nano materials science. Back in the late 1800s, Coley firstly treated sarcomas with a mixture of *Serratia marcescens* and *Streptococcus pyogenes* to reduce tumors and prolong survival of CRC patients (Ebrahimzadeh et al., 2021). Chiang and Huang (2021) developed a bacterial treatment for colorectal cancer using ECN to deliver therapeutic proteins and inhibits tumor growth. Kefayat et al. (2018) used alive attenuated *Salmonella Typhi* Ty21a as a vehicle for smart delivery of gold nanoparticles to the hypoxic regions of tumor and achieved high accumulation of folic acid functionalized gold nanoparticles.

Unique coating strategy of living bacteria could be need for special tumors on anti-tumor therapy. Intravenous injection could be the mainly delivery strategy of living bacteria on therapy of breast cancer. Coating materials should endow bacteria low immunogenicity to escape from immune attack rapidly into tumor sites for killing tumor cells. *In-situ* injection or subcutaneous injection adjacent to tumor is commonly used on bacteria-mediated anti-tumor on the solid melanoma. Synergistic antitumor materials with chemotherapy drug or photothermal therapy adjuvant could be suitable to coat living bacteria for efficient tumor cytotoxicity. Oral administration is an excellent strategy to deliver bacteria directly reach colorectal tumor through the GI tract. Therefore, the gastrointestinal protection, intestinal adhesion and inflammation targeting are key considerations for choosing coating materials of living bacteria on anti-tumor therapy.

## Conclusion and future perspectives

Bacterial therapy is a promising strategy for anti-tumor therapy due to its inherent tumor-targeting properties. Based on the natural tendency of tumor-targeted bacteria, various materials can be grafted onto the surface of bacteria by physical, chemical or biological methods to achieve a highly efficient and stable anti-tumor system by enhancing gastrointestinal stability, tumor tissue targeting, and tumor inhibition response. In this review, we have briefly summarized three kinds of materials used to coat bacteria in cancer therapy. Natural polymers could be easily acquired without a complex synthesis process. Synthetic materials could link with functionalized decoration on the surface of bacteria, hence achieving synergetic therapy on cancer. Cell-based materials show well biocompatibility and low immunogenicity to implement targeted delivery of bacteria on *in vivo* tumor. Tumor targeting bacteria has been applied on various tumors for diagnosis, imaging and treatment. The effective combination of bacterial therapy by functionalized encapsulation with other approaches is the current research hotspot, particularly immunotherapy and photothermal therapy. The intelligent application of bacterial tumor targeting could facilitate the development of sustainable bacteria-mediated therapies for routine clinical use.

Despite the attractive and promising therapeutic prospects, there are still many limitations and challenges restricting the development and application of bacteria-based delivery system. Safety is a major concern due to the immunogenicity of living bacteria. Higher microbe concentrations potentially could induce systemic toxicity (Din et al., 2016). The trial of *Bacillus Calmette-Guérin* or modified *Salmonella typhimurium* as medication in anti-tumor therapy is firstly carried out to validate their safety not therapeutic efficacy (Liang et al., 2019; Mukherjee et al., 2021). Therefore, the appropriate number of bacteria is essential to carry enough drugs ensuring both therapeutic effects and safety.

The manufacturing process of bacteria-based delivery system is more complex than that of small molecule anticancer drugs, which are different from traditional pharmaceutical processes. Therefore, a new methodology is needed for large-scale production, sterilization technology, storage and transportation of bacteria-based delivery products. Developing more effective and rational designs, bacteria-mediated therapies could be one of the most powerful tools against cancer in the future.
